# *Drosophila* Bestrophin-1 Currents Are Regulated by Phosphorylation via a CaMKII Dependent Mechanism

**DOI:** 10.1371/journal.pone.0058875

**Published:** 2013-03-12

**Authors:** Charity Duran, Li-Ting Chien, H. Criss Hartzell

**Affiliations:** Department of Cell Biology and Center for Neurodegenerative Disease, Emory University School of Medicine, Atlanta, Georgia, United States of America; Albany Medical College, United States of America

## Abstract

Cell swelling induced by hypo-osmotic stress results in activation of volume-regulated anion channels (VRAC) that drive a compensatory regulatory volume decrease. We have previously shown that the Best1 gene in *Drosophila* encodes a VRAC that is also activated by increases in intracellular Ca^2+^. The role of Best1 as a VRAC has recently been independently confirmed by the Clapham lab in an unbiased RNAi screen. Although dBest1 is clearly a volume-regulated channel, its mechanisms of regulation remain unknown. Here we investigate *Drosophila* Best1 (dBest1) regulation using the *Drosophila* S2 cell model system. Because dBest1 activates slowly after establishing whole-cell recording, we tested the hypothesis that the channel is activated by phosphorylation. Two experiments indicate that phosphorylation is required for dBest1 activation: nonspecific protein kinase inhibitors or intracellular perfusion with the non-hydrolyzable ATP analog AMP-PNP dramatically reduce the amplitude of dBest1 currents. Furthermore, intracellular perfusion with ATP-γ-S augments channel activation. The kinase responsible for dBest1 activation is likely Ca^2+^/calmodulin dependent kinase II (CaMKII), because specific inhibitors of this kinase dramatically inhibit dBest1 current activation. Neither specific PKA inhibitors nor inactive control inhibitors have effects on dBest1currents. Our results demonstrate that dBest1 currents are regulated by phosphorylation via a CaMKII dependent mechanism.

## Introduction

Volume-regulated anion channels (VRACs) are critical for cell volume homeostasis via a process called regulatory volume decrease (RVD) [1]–[3]. RVD is a process by which a cell returns to its normal volume after swelling in response to osmotic pressure differences across the plasma membrane. During RVD, activation of VRAC and other channels/transporters result in an efflux of ions followed by water, thereby returning the cell to its normal volume. Several molecular candidates have been proposed to mediate VRAC [4], [5]. Best1 is one such candidate that has received considerable support for being a VRAC in *Drosophila* S2 [6]–[9] cells.

We have previously shown that the dBest1 gene in *Drosophila* S2 cells encodes an anion channel. The dBest1 current can be activated by increases in intracellular Ca^2+^ and is abolished by RNAi directed against dBest1 [6]. The dBest1 current is also activated by extracellular hypo-osmotic solutions and thus is a candidate for the volume-regulated anion channel (VRAC) in these cells. Evidence in support of this hypothesis is that the VRAC current was abolished by RNAi directed against dBest1 [7]. Furthermore, cells with dBest1 expression knocked down by RNAi fail to undergo RVD in response to cell swelling. The effect of Best1 RNAi was rescued by over-expression of wild type dBest1 as well as a mutant dBest1 that had altered anion selectivity [8]. These experiments showed conclusively that the VRAC current was mediated by dBest1. Recently, Stotz et al. [9] confirmed our conclusions. They performed an unbiased genome-wide RNAi screen to identify the VRAC channel in S2 cells and concluded that Best1 was the most likely candidate.

Cell volume and Ca^2+^ may independently regulate dBest1 function, because Ca^2+^ can activate the current in the absence of cell volume changes and increases in cell volume can activate the current even when intracellular Ca is highly buffered. In general, the mechanisms underlying ion channel regulation by cell volume are very complex, and multiple signaling pathways have been implicated [2], [3]. Thus, it is unknown if cell volume and Ca^2+^ converge on a common regulatory pathway to activate dBest1, as very little is known about mechanisms that contribute to dBest1 channel regulation. Although human Best1 does not seem to require phosphorylation for activation, it is modulated by phosphorylation [10]–[12]. Here we examine the requirement for phosphorylation in dBest1 activation using whole cell patch clamp recording of *Drosophila* S2 cells.

## Materials and Methods

### Cell Culture

*Drosophila* S2 cells were obtained from the Drosophila Genomics Resource Center (Indiana University) and cultured at room temperature (22–24°C) in Schneider’s *Drosophila* Medium (GIBCO BRL) containing 10% heat-inactivated FBS (GIBCO BRL), 50 µg/ml penicillin, and 50 µg/ml streptomycin (GIBCO BRL). HEK293 cells (ATCC) were cultured in DMEM supplemented with 10% FBS and 0.5% penicillin-streptomycin at 37°C.

### Transfection

HEK293 cells were transfected using Fugene 6 (Roche) with 1 µg plasmid DNA. For electrophysiological recordings, dAno1 cloned from S2 cells (NM_142563.1) was subcloned into the pIRES2-EGFP vector (Clontech). Transfected cells were plated at a low density and used for electrophysiology 24–48 hrs after transfection. Cells expressing GFP were patched.

### Electrophysiology

Whole cell patch-clamp was performed at room temperature (22–24°C). Patch pipettes were fire polished to resistances of 2–3 MΩ. The standard extracellular solution used for patch clamping S2 cells contained (in mM) 150 NaCl, 2 CaCl_2_, 1 MgCl_2_, HEPES (pH7.2 with NaOH), and 10 glucose. The standard pipette solution contained 143 CsCl, 10 Ca-EGTA-NMDG, 8 MgCl_2_, 10 HEPES (pH 7.2 with NMDG), 10 glucose, and 3 ATP. The free Ca^2+^ concentration for this solution was ∼4.5 µM. Osmolarity of both extracellular and intracellular solutions was adjusted to 320 mOsm with water or sucrose. For HEK293 cell recordings, the intracellular solution contained 146 CsCl, 2 MgCl_2_, 5 Ca-EGTA (∼24 µM free Ca^2+^), 10 sucrose, and 8 HEPES, pH 7.3 with NMDG. The extracellular solution contained 140 NaCl, 5 KCl, 2 CaCl_2_, 1 MgCl_2_, 15 glucose, and 10 HEPES, pH 7.4 with NaOH. Osmolarity was adjusted with sucrose to 303 mOsm. Cells were voltage clamped with ramps from −100 mV to +100 mV run at 10-s intervals, followed by a voltage step protocol from −100 to +100 mV.

### Pharmacology

ATP-γ-S, AMP-PNP, H7-dihydrochloride, calyculin A, K252a, KN-62, KN-93, and staurosporine (STP) (Sigma-Aldrich) were made as stock solutions and diluted to their final concentrations in extracellular and intracellular recording solutions. ATP analogs were included in the patch pipette solution. Kinase inhibitors were included in both the bath and patch pipette for whole-cell recordings. S2 cells were exposed to extracellular solution containing drug for at least 6 minutes prior to recording. Cells were incubated with myristoylated-AIP for 20 min prior to recording due to its relative impermeability to the membrane.

### Data Analysis

Data were analyzed using pClamp 8.2 software and Origin 7.0. A Student’s t-test was performed to determine significant differences in maximum current between control and drug treated cells (p<0.05). Data is expressed as mean +/− SEM.

### Phosphorylation Site Prediction

Phosphorylation sites were predicted using either Phosphomotif Finder (www.hprd.org/PhosphoMotif_finder) or GPS2.1 (http://gps.biocuckoo.org/). Accession numbers for the dBest1 and hBest1 sequences analyzed are AAL29094 and AAH41664.1, respectively.

## Results

### *Drosophila* Anoctamins are Minor Contributors to Endogenous S2 Cell CaCCs

Previous studies demonstrate that endogenous CaCC currents in *Drosophila* S2 cells are mediated by dBest1 [6]–[9], but the more recent discovery that anoctamins function as CaCCs raises the possibility that *Drosophila* anoctamin orthologs also contribute to these currents. To address this issue, we identified *Drosophila* anoctamin orthologs using a Flybase blast search (http://flybase.org) for which we used the polypeptide sequence for human Ano1 as the input (NP_060513.5). Several *Drosophila* anoctamin orthologs were identified: *CG16718, CG6938, CG10353, CG15270, and Axs*. Of the genes identified, *CG16718* had the highest sequence similarity to hAno1. Because several Ano1 orthologs have been clearly shown to function as CaCCs, we chose to more closely examine the channel function of *CG16718*, which will be referred to as dAno1 for the purpose of this study.

**Figure 1:**
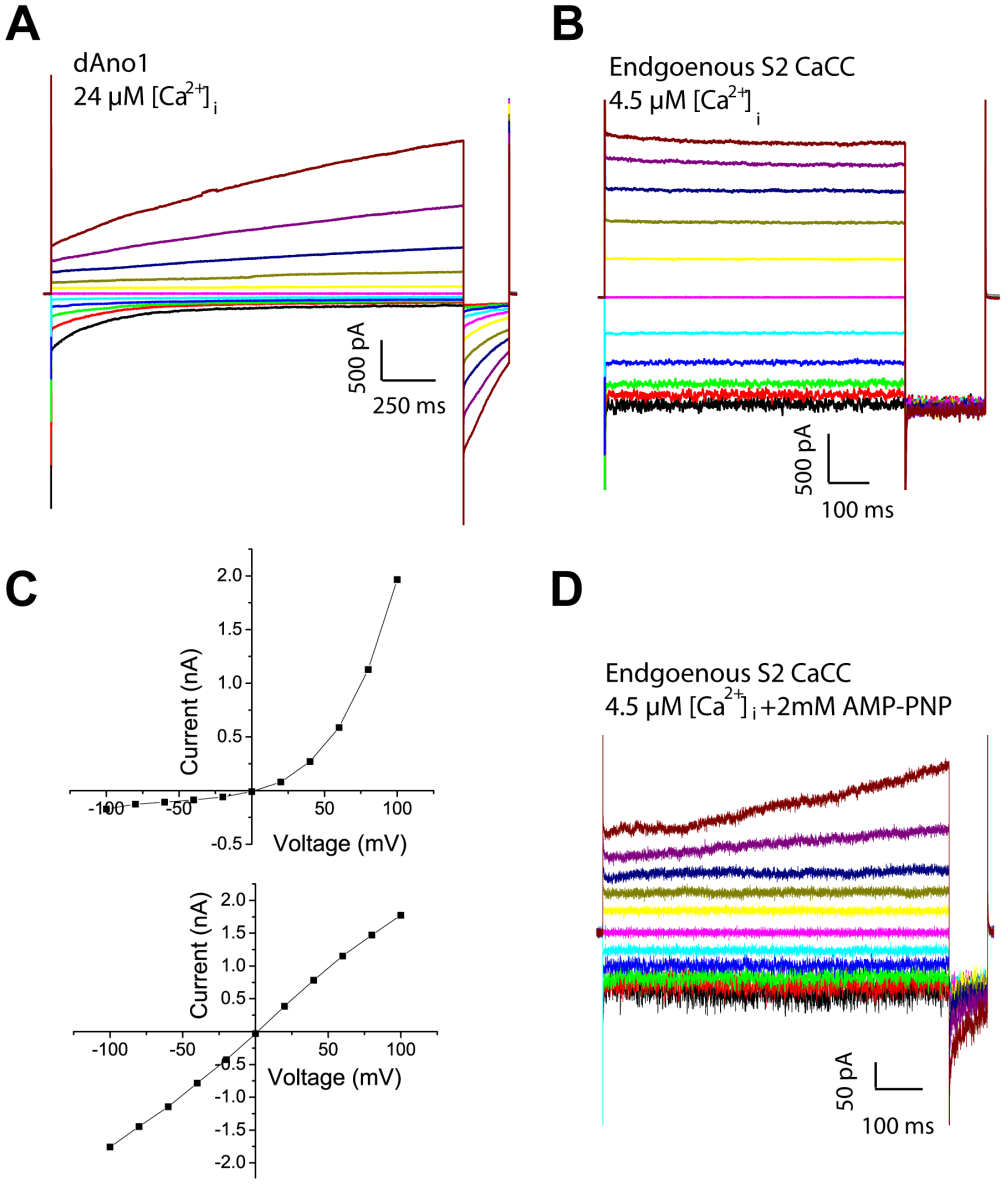
Comparison of endogenous S2 CaCC with heterologously expressed dAno1. **A)** Whole-cell recording from HEK cell expressing dAno1 using voltage steps from −100 mV to +100 mV applied in 10 sec intervals. **B)** Endogenous S2 cell CaCC current response to voltage steps from −100 mV to +100 mV in the presence of 4.5 µM intracellular Ca^2+^. **C)** IV curves from A (top) and B (bottom). **D)** Representative recording of endogenous S2 cell CaCC current in the presence of 2 mM AMP-PNP in the intracellular solution. The current is largely blocked, but a small Ano1-like current remains.

dAno1 (NM_142563.1) cloned from S2 cells was expressed in HEK293 cells to examine its channel function. Expression of dAno1 generated currents with biophysical properties distinct from those of the endogenous dBest1 current. Unlike dBest1, dAno1 currents strongly outwardly rectify and exhibit tail currents upon repolarization to −100 mV from positive voltages (Fig. 1A). In addition, dAno1 currents are time dependent and activate slowly. In contrast, endogenous dBest1 currents exhibit a linear I-V relationship and little or no time dependence (Fig. 1B, C). Furthermore, dBest1 expression in HEK293 cells generates currents biophysically similar to those of endogenous S2 CaCCs [6]. In a minority of S2 cells, a small (<300 pA) dAno1-like current was revealed when we included intracellular AMP-PNP, an ATP analog which inhibits the activation of dBest1 currents (shown below) (Fig. 1D). Given that endogenous S2 CaCC currents are ∼10-times larger in amplitude, and have biophysical properties that closely resemble dBest1, dAno1is unlikely to contribute significantly to endogenous CaCCs in S2 cells. Therefore, S2 cells are an ideal model for studying dBest1 function.

### dBest1 Currents are Regulated by Phosphorylation

**Figure 2:**
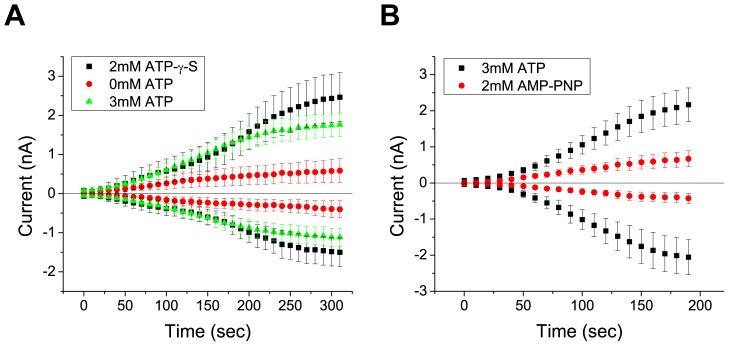
Effect of ATP and ATP analogs on time-dependent activation of S2 dBest1 Cl^−^ currents in response to high [Ca^2+^]_i_. dBest1 currents were measured by 1-sec duration voltage ramps from −100 mV to +100 mV. The figures plot the amplitudes of the ouward currents measured at +100 mV and inward currents measured at −100 mV. **A)** Average current response of *Drosophila* S2 cell Ca^2+^-activated dbest1 currents with high Ca^2+^ (4.5 µM) in the presence of 3 mM ATP, 0 mM ATP, or ATP-γ-S in the patch pipet. **B)** Activation of *Drosophila* S2 cell Ca^2+^-activated dBest1 currents with 3 mM intracellular ATP, or 3 mM ATP with 2 mM AMP-PNP. Error bars indicate mean +/− SEM. N = 6–8 for all conditions.

**Figure 3:**
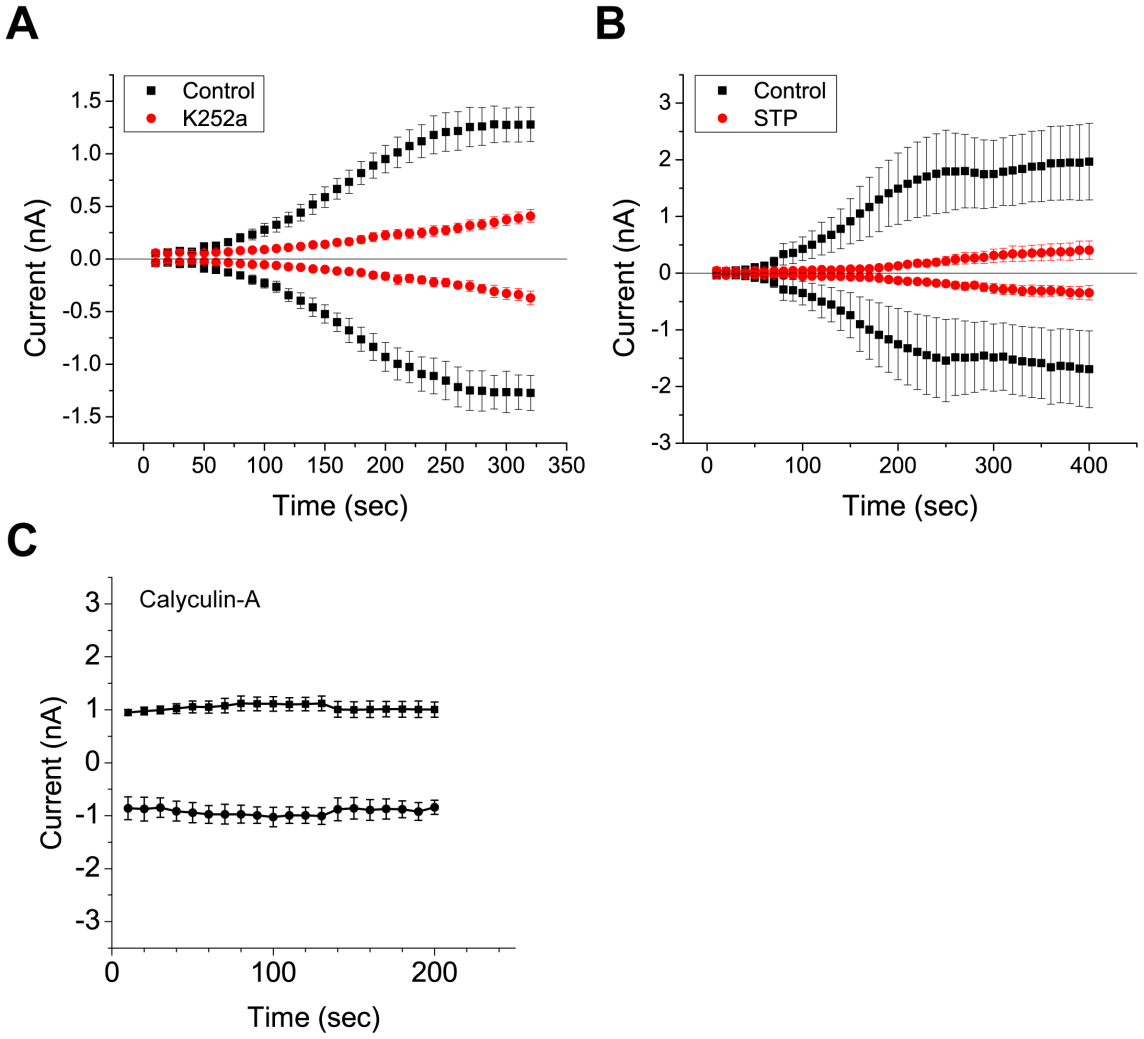
Effects of nonselective kinase/phosphatase inhibitors on *Drosophila* S2 Ca^2+^-activated currents. Cells were pre-incubated with either k252a, staurosporine, or calyculin A before being recorded with pipette solutions containing the inhibitor in addition to high Ca^2+^ and ATP. dBest1 currents are inhibited by **A)** k252a (1 µM) (n = 6) and **B)** staurosporine (10 µM) (n = 5). **C)** Application of 1 µM calyculin A results in currents that are stable and do not run-up over time. Error bars indicate mean +/− SEM.

*Drosophila* Best1 currents are activated by increases in intracellular Ca^2+^. However, the mechanisms underlying Ca^2+^ regulation of dBest1 are unknown. Two basic mechanisms could explain activation of dBest1 by Ca^2+^. Ca^2+^ could bind directly to dBest1 (or an accessory subunit) to induce a conformational change which gates the channel open. Alternatively, Ca^2+^ could bind to and activate Ca^2+^-dependent protein kinases or protein phosphatases, which regulate dBest1 activation via phosphorylation. Because dBest1 activates slowly over a period of minutes after establishing whole cell recording with high Ca^2+^ in the internal solution [6], we hypothesized that current activation requires phosphorylation. To test this idea, we first performed whole cell patch clamp in the presence or absence of intracellular ATP. With high intracellular Ca^2+^ (4.5 µM) and 3 mM ATP, dBest1 currents activate slowly over a period of 4–5 min before reaching a plateau (Fig. 2A). Exclusion of ATP from the intracellular solution significantly decreases the rate of Ca^2+^-dependent current activation (Fig. 2A). The slow time course of activation of dBest current and its facilitation by intracellular ATP suggests that phosphorylation is involved in dBest1 activation. To further test this hypothesis, we examined the effect of ATP analogs on dBest1 current activation. Substitution of ATP with ATP-γ-S, an ATP analog that yields thiophosphorylated proteins resistant to protein phosphatases, augmented the currents slightly. Conversely, inclusion of AMP-PNP, a non-hydrolyzable ATP-analog and competitive inhibitor of phosphorylation, significantly decreased the amplitude of dBest1 currents, further implicating phosphorylation in dBest1 activation (Fig. 2B). We next examined the effects of non-specific protein kinase/phosphatase inhibitors on dBest1 currents (Fig. 3). K252a and staurosporine are potent nonselective protein kinase inhibitors that bind to the ATP binding site of many protein kinases. When added to the bath and pipette solutions, both K252a and staurosporine caused a dramatic slowing of dBest1 current activation by Ca^2+^ and a decrease in the maximal amplitude of Ca^2+^-activated dBest currents (Fig. 3A, B). Conversely, application of calyculin A, a serine/threonine phosphatase inhibitor (Fig. 3C), yielded currents that were 1 nA immediately upon achieving whole-cell recording. Unlike control currents, these currents did not run-up over time and remained stable. These data support a requirement of phosphorylation in dBest1 activation.

### Regulation of dBest1 Currents by Phosphorylation is CaMKII-dependent

**Figure 4:**
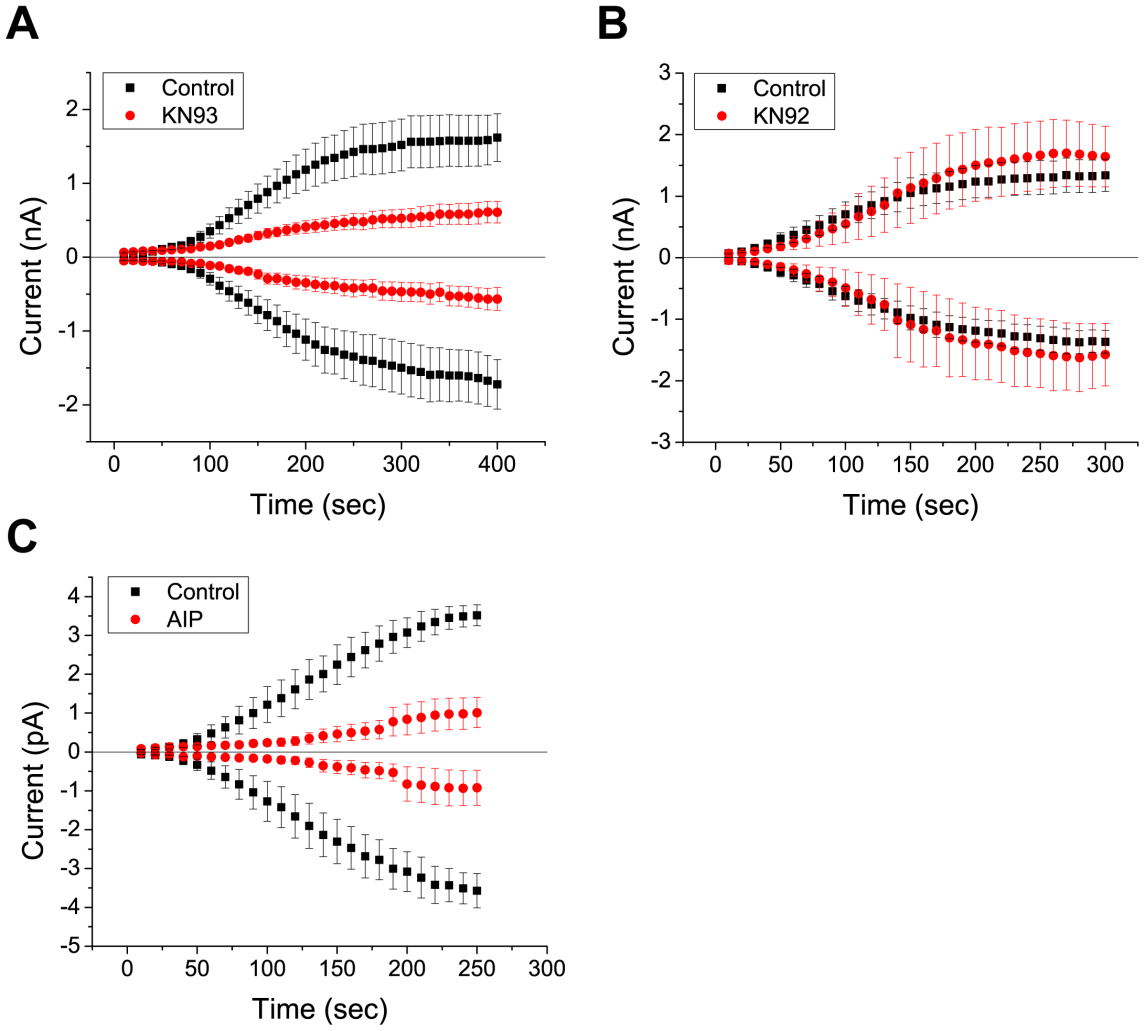
*Drosophila* Ca^2+^-activated dBest1 currents are inhibited by a potent CaMKII inhibitor, KN-93. **A)** KN-93, (10 µM), reduces current amplitude, whereas its inactive structural analogue, **B)** KN-92 (10 µM), has no effect on dBest1 currents. **C)** A very specific peptide inhibitor of CAMKII, myristoylated AIP, also inhibits dBest1 currents. Error bars indicate mean +/− SEM.

**Figure 5:**
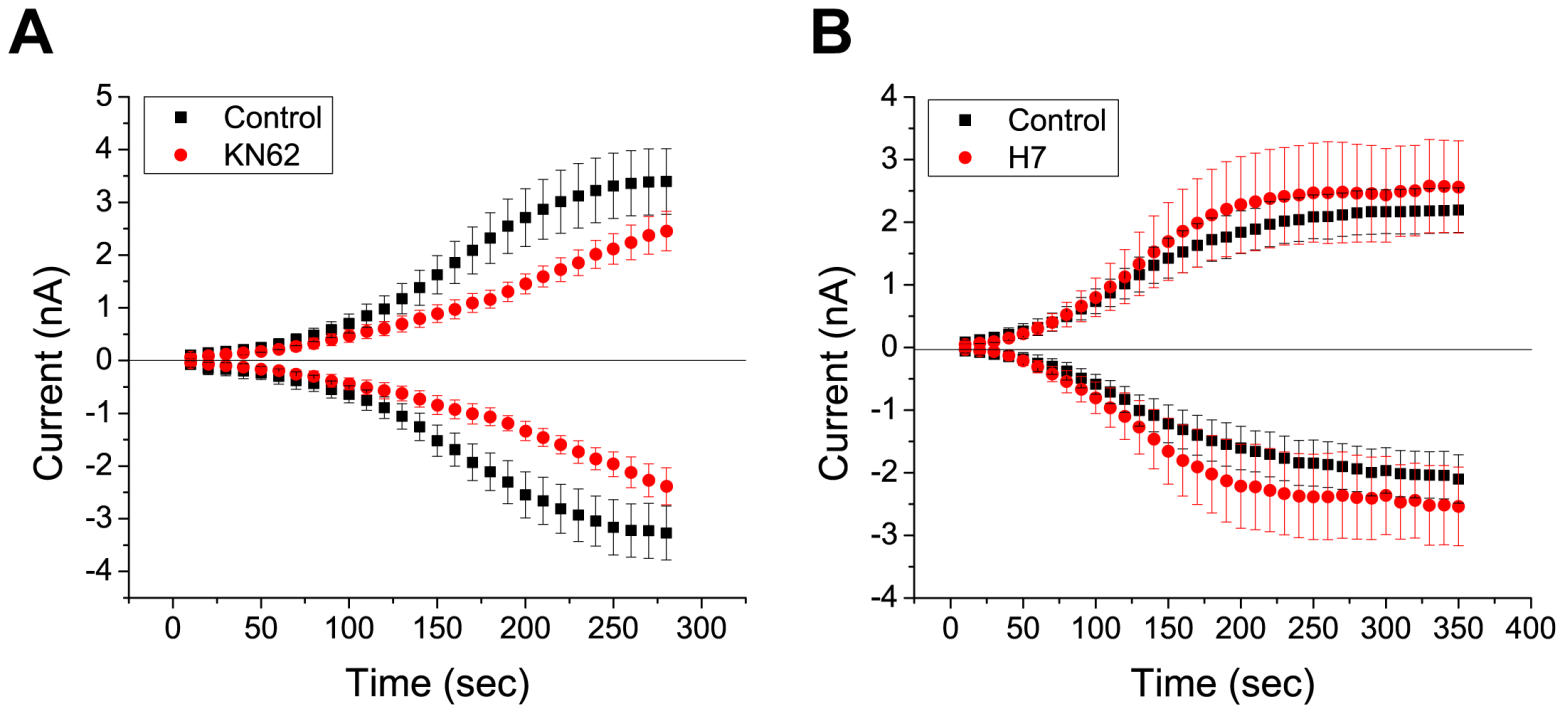
Inhibitors of PKA have no effect on dBest1 currents. **A)** KN-62 (10 µM) also does not have a significant effect on dBest1 currents. KN-62 is a relatively less potent CAMKII inhibitor than KN-93. Error bars represent mean +/− SEM.**B)** H7 (10 µM), which selectively inhibits PKA at this concentration, has no effect on dBest1 currents.

**Figure 6:**
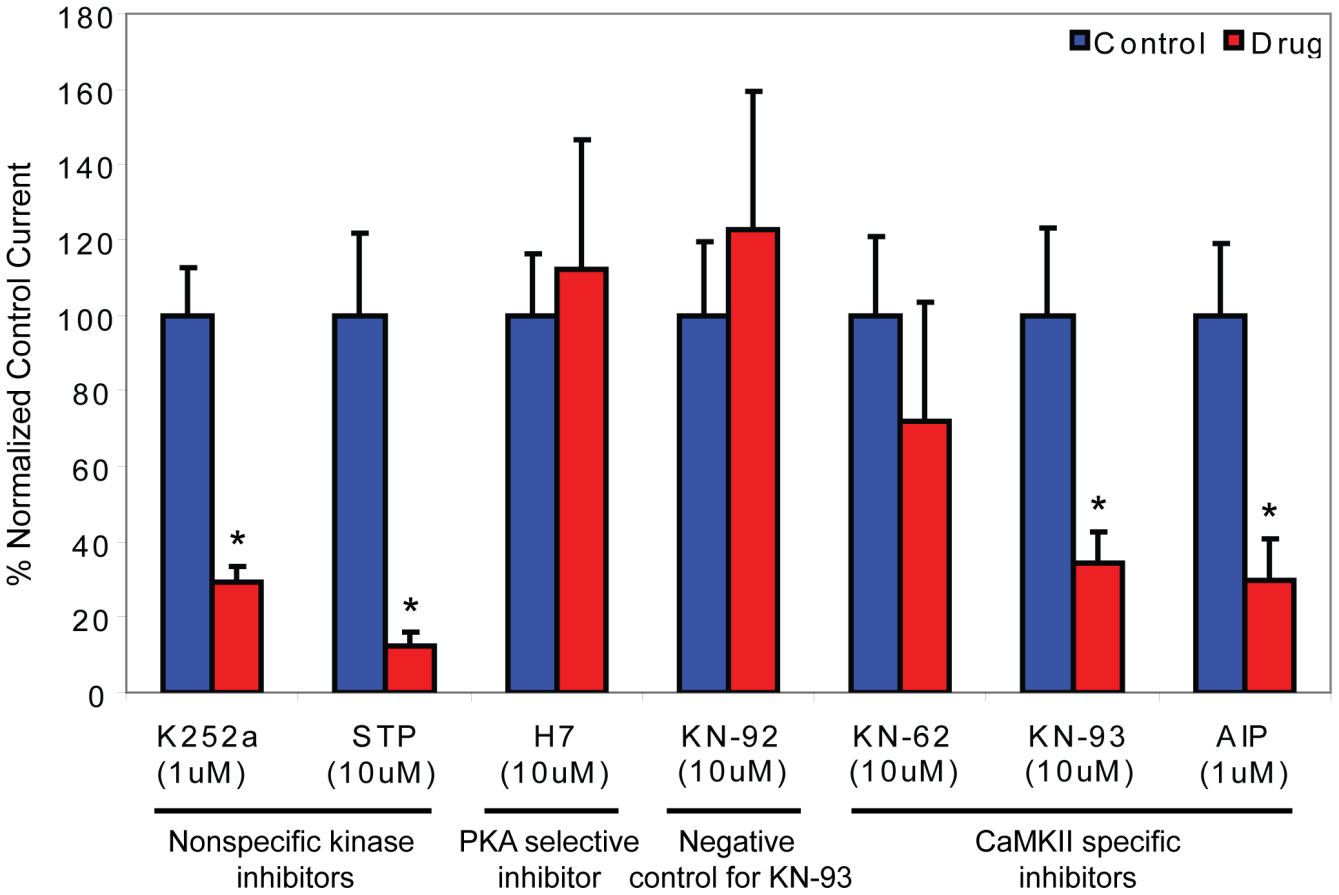
Comparison of the effects of various kinase inhibitors. Currents were measured at 100 mV 300-s after break-in. Data represent the percentage of the normalized control current. Error bars indicate mean +/− SEM. Asterisks denote currents that are significantly different from control currents as determined by student’s t-test (p<0.05). N = 5–8 for all conditions.

We next set out to determine which specific kinases regulate dBest1 activity. We examined the effect of CaMKII inhibitors on dBest1 currents (Fig. 4). Application of KN-93, a selective and potent CaMKII inhibitor [13], significantly reduced dBest1 current amplitude (Fig. 4A). An inactive structural analog of KN-93, KN-92, had no effect on dBest1 currents (Fig. 4B). These results indicate that the effects of KN-93 are mediated through inhibition of CaMKII, rather than through nonspecific effects of the drug. A lower potency CaMKII inhibitor, KN-62, slightly decreased dBest1 current amplitude (Fig. 5A); however, it did not reduce currents to the same extent as KN-93. KN-62 is ∼4-times less potent than KN-93 in inhibiting CaMKII [13], [14]. Myristoylated autocamtide-2 related inhibitory peptide (AIP), a highly-specific and potent inhibitor of CaMKII [15], dramatically inhibited dBest1 currents. To determine whether other protein kinases might also be involved, and to confirm the specificity of the effects of the CaMKII inhibitors, the effects of a specific inhibitor of cAMP-dependent protein kinase (PKA), H7, was tested (Fig. 5B). This inhibitor had no effect on dBest1 current amplitude. The effects of various kinase inhibitors on dBest1 current are quantified in Fig. 6.

## Discussion

### dBest1 Currents are Regulated via a CaMKII Dependent Mechanism

**Figure 7:**
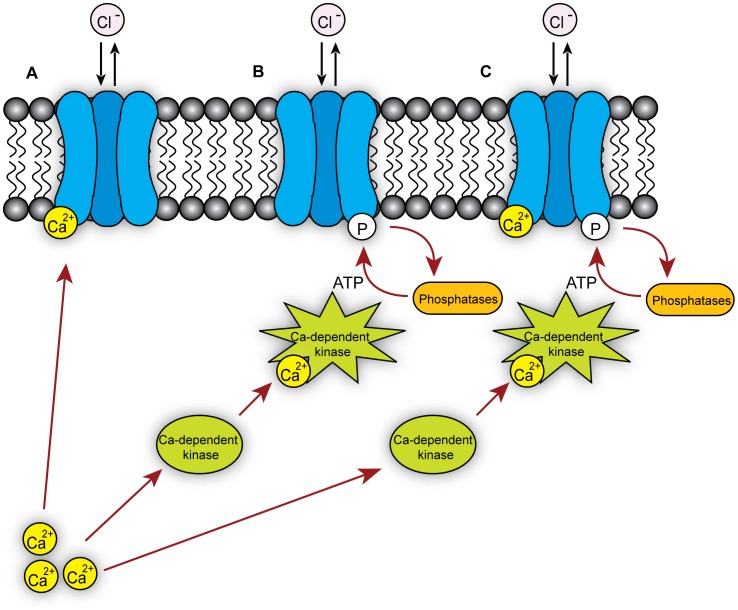
Models of *Drosophila* Bestrophin 1 mechanisms of activation/regulation. dBest1 can be activated **A)** by Ca^2+^ alone, **B)** by Ca^2+^-dependent protein kinases, or **C)** by both Ca^2+^ and Ca^2+^-dependent protein kinases.

The effects of various kinase inhibitors on dBest1 reveals that current activation by intracellular Ca^2+^ is dependent on protein phosphorylation, which is primarily mediated via CaMKII. We cannot rule out the contribution of other kinases or phosphatases, but cAMP-dependent phosphorylation does not seem to be involved. Potential models for dBest1 regulation by Ca^2+^ [6], CaM-dependent kinases, and phosphatases [10] are summarized in Fig. 7.

### Potential CaMKII Phosphorylation Sites

**Figure 8:**
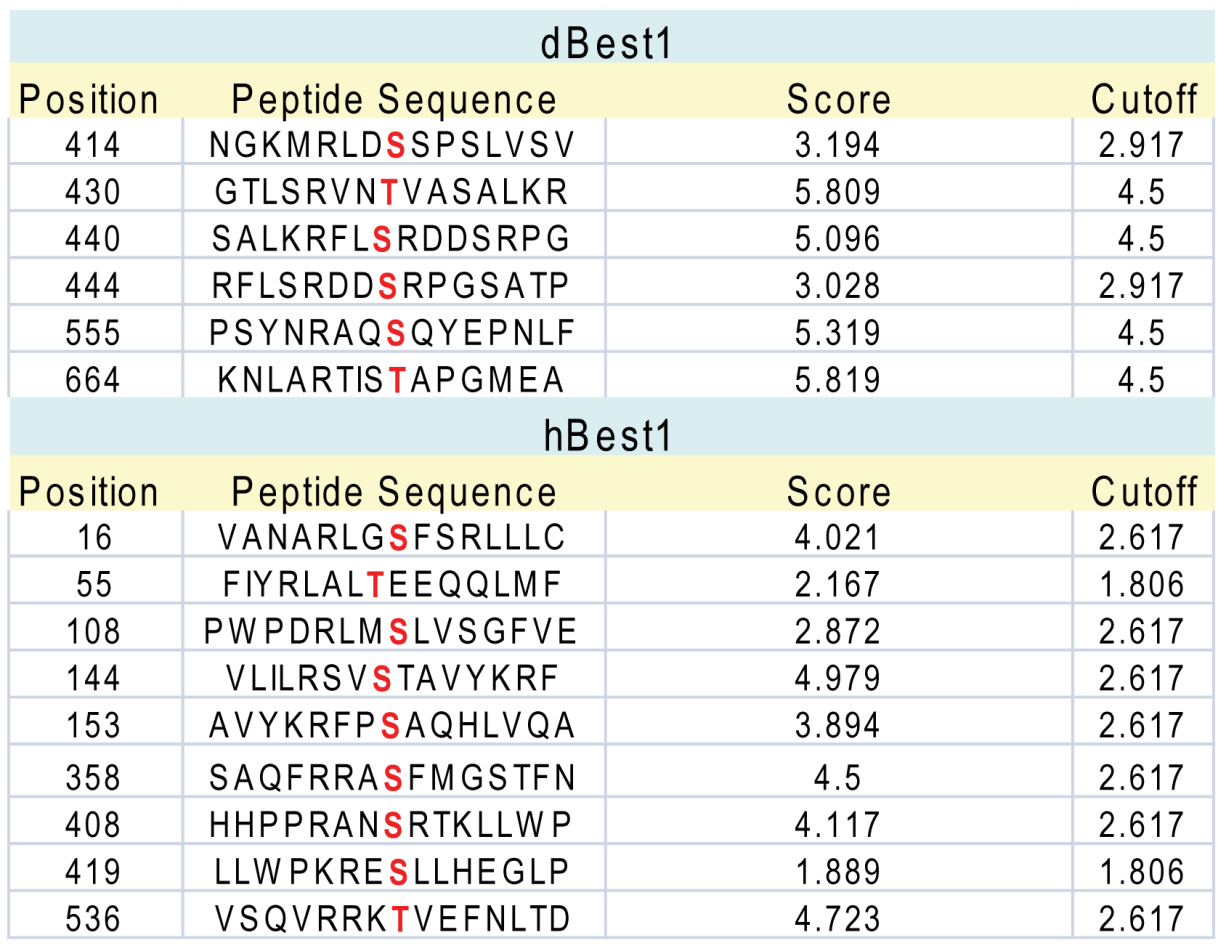
Potential CaMKII phosphorylation sites in dBest1 identified by GPS 2.1. Predicted CaMKII phosphorylation sites within dBest1 (AAL29094.1) and hBest1 (AAH41664.1) are indicated in red.

We used two different phosphorylation site prediction programs to determine regions potentially implicated in regulation of dBest1 by CaMKII. Although prediction algorithms are notoriously unreliable, they can provide guidance to future studies. We used GPS2.1 which has a greatly improved algorithm to reduce false positives [16]. Many of the predicted CaMKII phosphorylation sites are located in the C-terminus, which we and others have shown is very important in human Best1 function (Fig. 8). We also used another phosphorylation site identification program, Phosphomotif finder [17], which does not employ algorithms or computational strategies to predict phosphorylation, but rather reports the presence of any literature-derived motifs. Phosphomotif finder identified many of the same potential CaMKII phosphorylation sites as GPS2.1.

Human Best1 has been shown to be phosphorylated by PKC [2]. The serine-358 phosphorylation site, which lies within the C-terminus of hBest1, may participate in regulation of hBest1 currents by hypertonic stress [2]. Phosphorylation of S358 by PKC slows channel rundown during whole-cell recording, while dephosphorylation via PP2A accelerates channel rundown [10], [11]. The effect of PKC or PP2A inhibitors on dBest1 current was not examined here. However, S358 is not conserved in dBest1, suggesting that dBest1 and hBest1 are probably differentially regulated by phosphorylation.

Given the importance of the C-terminus in the regulation of hBest by phosphorylation, one wonders whether a CaMKII phosphorylation site may be conserved between human and *Drosophila* Best1. Although there are several predicted CaMKII phosphorylation sites located in the same general region of the sequence alignment (for example hBest1 S419 and dBest1 T430), the C-terminus of hBest1is sufficiently divergent from dBest1 that it is not possible to predict whether these amino acids have any homology. Furthermore, it is unknown if CaMKII directly phosphorylates dBest1, or if there are intermediate signaling events involved in dBest1 activation by CaMKII.

Although CaMKII is critical for dBest1 activation, it is unknown if direct Ca^2+^ binding is also important for activation of dBest1. We previously reported that hBest1 is activated by direct Ca^2+^ binding to an EF hand at position 312–323, located within the C-terminus of hBest1 [11]; this EF hand is fully conserved in dBest1, suggesting a role for direct Ca^2+^ binding in dBest1 activation. For hBest1, activation by Ca^2+^ binding to the EF hand also requires an adjacent acidic amino acid rich regulatory region at position 350–390, which also plays a role in channel rundown. However, several of the acidic residues in this region are not conserved in dBest1. Interestingly, many of the predicted CaMKII phosphorylation sites fall near this regulatory region in a stretch of amino acids that are unique to dBest1. Although speculative, it is possible that the phosphorylation state of this region is important for regulation/activation of dBest1 by Ca^2+^. Taken together, these findings support the model of channel activation presented in Fig. 6C, in which both Ca^2+^ binding and Ca^2+^-dependent phosphorylation by CaMKII are critical for channel activation.

In the literature, there are many examples of endogenous CaCC currents (whose molecular identities remain uncertain) that are regulated by CaMKII [18]. Regulation of CaCCs by CaMKII is cell-type dependent, because it has been reported that CaMKII may have an inhibitory or stimulatory effect on endogenous CaCC currents [18], [19]–[22]. It is important to note, however, that these differences are likely explained by heterogeneity in the proteins that underlie endogenous CaCCs in these systems, as some are encoded by anoctamins and others by bestrophins or possibly other genes.

### Future Directions

These preliminary studies provide future directions for examining the regulatory mechanisms of Best1. The foremost question posed by this study is: can dBest1 be directly phosphorylated, and if so, is phosphorylation directly mediated by CaMKII? Alternatively, if dBest1 is not directly phosphorylated by CaMKII, CaMKII may act on a regulatory subunit of dBest1. There are several possible mechanisms for how phosphorylation could regulate dBest1 activity. First, the phosphorylation state of dBest1 may directly regulate its channel function, as has been shown for hBest1. Phosphorylation of dBest1 could also alter its trafficking to the plasma membrane. Or, phosphorylation of dBest1 could affect its association with regulatory subunits, and vice versa.

Although the mechanisms underlying dBest1 regulation by phosphorylation are uncertain, it is clear that CaMKII is important for activation of dBest1 by intracellular Ca^2+^. It will be interesting to see if CaMKII is also critical for volume dependent activation of dBest1, and if this regulatory mechanism is conserved in hBest1. Inhibition of hBest1 by hypertonic stress was previously shown to be dependent on its phosphorylation state [12]. Therefore, these results provide a basis for studying the role of CaMKII in hBest1 regulation by hypertonic stress, and RVD.
